# Perceptions surrounding the possible interaction between physical activity, pollution and asthma in children and adolescents with and without asthma

**DOI:** 10.1186/s12889-023-17174-6

**Published:** 2023-12-05

**Authors:** Kathryn A. Jordan, Kelly A. Mackintosh, Gwyneth A. Davies, Chris J. Griffiths, Paul D. Lewis, Melitta A. McNarry

**Affiliations:** 1 Department of Sport and Health Sciences, Applied Sports, Technology, Exercise and Medicine (A-STEM) Research Centre, Swansea University, Swansea, Wales UK; 2 Swansea University Medical School, Swansea University, Swansea, Wales UK; 3 Barts Institute of Population Health Sciences, Queen Mary University of London, London, UK; 4 School of Management, Swansea University and Vindico ICS, Swansea, UK

**Keywords:** Air pollution, Physical activity, Asthma, Children

## Abstract

A cornerstone of asthma management is maintaining physical activity (PA), but this may lead to increased exposure to, and deeper inhalation of, pollutants. Furthermore, children and adolescents may be more susceptible to the deleterious impacts of such exposures. Despite the recent air quality campaigns and media coverage surrounding the dangers of air pollution to respiratory health, few target children and their understanding of such issues.

Using semi structured interviews, understanding of PA, air pollution and their interaction was explored with 25 youth aged 7—17 years. Utilising NVIVO 12 software, an atheoretical, inductive thematic analysis was conducted to identify key themes which were subsequently presented as pen profiles with the number of common responses within a theme indicative of its strength.

The majority (88%) of youth’s indicated traffic-related air pollution and global manufacturing as key sources of air pollution. Whilst all youths were aware of outdoor pollution, only 52% were aware of indoor air pollutants, of which 62% had asthma. Despite some uncertainty, all youths described pollution in a negative fashion, with 52% linking air pollution to undesirable effects on health, specifically respiratory health. PA in a polluted area was thought to be more dangerous than beneficial by 44%, although 24% suggested the benefits of PA would outweigh any detriment from pollution.

Youth are aware of, and potentially compensate for, the interaction between air pollution and PA. Strategies are needed to allow youth to make more informed decisions regarding how to promote PA whilst minimising exposure to air pollution.

## Background

Air pollution is a global problem, responsible for an estimated seven million deaths per year and is the single largest environmental health risk [40]. Indeed, air pollution is associated with increased airway sensitivity, mucosal damage, impaired mucus clearance and increased allergen penetration [11, 17, 35]. These deleterious effects are exacerbated in individuals with underlying respiratory conditions who are at an increased risk of developing symptoms that require medical intervention [8]. People with asthma are recognised as being particularly at risk from increased exposure to air pollution, with evidence of impaired lung function, increased exhaled nitric oxide (FeNO), and an increased risk of exacerbation and hospitalization [10, 17]. Furthermore, a recent meta-analysis showed that exposure to traffic-related air pollution (TRAP) was positively correlated with the development of childhood asthma [21].

More recent research suggests that air pollution plays a role in increasing oxidative stress with can lead to a heightened immune response, and subsequently the development of autoimmune conditions and airway sensitivity [41]. Increased oxidative stress and systemic inflammation could lead to airway damage and, indeed, asthma. However, physical activity (PA) is well established to have beneficial effects on the respiratory system, including being associated with an increase in aerobic fitness and lower incidence of exercise-induced bronchospasm (EIB) in those with asthma [28]. However, it has been questioned whether these benefits may be counteracted by an increased exposure to, and deeper inhalation of, air pollution during PA [21]. Perhaps unsurprisingly, the reported incidence and severity of asthma in children is greater in inner cities [21]. Being active in urban settings increases the exposure to particulates, and at a higher concentration. Given that PA is widely advocated as a management strategy for those with asthma, it is important to understand this complex relationship.

Children may be more susceptible to the adverse effects of air pollution than adults [14]. It is believed this is the related to numerous factors, including a higher respiratory rate [20], a greater physiological sensitivity to pollutants [11], more time being spent outdoors [14], and a tendency to breathe through the mouth, thus rendering the nasal filtering system ineffective. There is little information currently available regarding the interactions between air pollution, lung function, age and maturation. However, the long maturational process of the respiratory system means that childhood represents a critical exposure period for the development of asthma [29], thereby potentially compounding the sensitivity to air pollution, which is further compounded by a higher air to body weight intake than adults [2]. Indeed, a key pathological characteristic of asthma is the narrowing of the airways [3], which may further exacerbate the effects of air pollution. An immature immune system, coupled with a developing respiratory system is believed to put children at increased risk from air pollution, especially when exercising outdoors [38]. Furthermore, the American Academy of Pediatrics (2021) reported that children who attended a school or lived in an area of high TRAP (traffic related air pollution) had reduced lung function and increased incidence of respiratory symptoms.

Despite increased awareness of the potential health implications associated with air pollution exposure, little is known regarding whether children, irrespective of asthma status, perceive it as a potential barrier to PA. This may be due, at least in part, to a conventional perception that children struggle to recognize patterns and interrelationships and rationalize environmental systems [4]. However, given the recent increase in media coverage and number of campaigns targeted specifically at reducing air pollution, it is reasonable to expect an increased understanding. Children are therefore likely to start recognizing the role of the physical environment at a younger age. Therefore, the aim of this study was to determine children and adolescents’ perceptions regarding asthma, air pollution, PA and their interaction. This is vital in order to develop appropriate, acceptable and sustainable interventions to increase PA among young people, particularly those with asthma, in a safe environment, when air pollutants are above recommended levels.

## Methods

### Study design and participants

This study was a cross-sectional, qualitative study, using individual semi-structured interviews to explore the beliefs of children and adolescents aged between 7 and 17 years. The inclusion of both primary and secondary aged children was deemed appropriate based on research [4] which showed children are capable of understanding their environment earlier than many studies consider, and in an attempt to capture growing understanding with age. Participants were recruited via the social media platforms “Facebook” and “Instagram”, between July and December 2020, with 32 families expressing an interest in participating. Following informed parental/guardian consent and participant assent, 25 interviews were conducted online, typically lasting no longer than 1-h (38 min ± 10 min), and with consent and assent confirmed verbally prior to the commencement. Ethics approval was granted by Swansea University and the study was conducted in accordance with the Declaration of Helsinki (2013).

Anthropometrics were recorded remotely by parent’s/care givers due to the COVID-19 pandemic, and are described in Table 1. Of those participating, 11 reported a doctor diagnosis of asthma, whilst 14 described themselves as healthy. Ethnicity, location, age, academic achievement estimates and socioeconomic status (SES) according to indices of multiple deprivation were recorded. The English Index of Multiple Deprivation (IMD) 2019, the Welsh Index of Multiple Deprivation (WIMD) 2019, the Scottish Index of Multiple Deprivation (SIMD) 2020 and the Northern Ireland Index of Multiple Deprivation Measure (2017) were used as appropriate which accounted for parental education, home postcode, income, employment, health, crime, barriers to housing and living environment when determining SES.

**Table 1:** Participant characteristics

	Mean ± SD	Asthma *(n* = *2)*	Non-Asthma *(n* = *7)*
**Primary school**			
**Age (years)**	8.89 ± 1.83	9.50 ± 2.12	8.71 ± 1.89
**Weight (kg)**	36.01 ± 6.70	40.55 ± 6.29	34.79 ± 6.68
**Height (m)**	1.42 ± 0.14	1.50 ± 0.08	1.40 ± 0.15
**Secondary school**		*(n* = *9)*	*(n* = *7)*
**Age (years)**	13.94 ± 1.84	14.13 ± 1.64	13.75 ± 2.12
**Weight (kg)**	53.85 ± 18.52	58.93 ± 11.58	48.03 ± 23.89
**Height (m)**	1.65 ± 0.11	1.70 ± 0.11	1.62 ± 0.09

### Interviews

Participants were interviewed remotely using video conferencing software (Zoom Video Communications Inc.) which did not require participants to download the program or create a profile. The application allowed the inclusion of participants from diverse geographical locations and ensured the safety of all those involved during the COVID-19 pandemic. Interviews were recorded using the embedded feature, and subsequently transcribed verbatim by the lead researcher. Privacy and confidentiality were assured, with only the lead researcher having access to the videos, with all information stored secured on their password protected laptop. All interviews were conducted by the first author (KAJ), who holds a valid teaching qualification.

Participants underwent a semi-structured individual interview, with parents and caregivers asked not to influence the child’s responses, or comment during the interview. Children were asked to define and describe their understanding of the three key themes of PA, air pollution and asthma. Three primary school aged children expressed confusion over the term ‘physical activity’ and thus a succinct and short definition was provided for them (i.e., “physical activity is when we are moving our body”). The interviewer confirmed understanding by asking the child to identify types of activities this would include. All children and adolescents answered the same set questions, irrespective of age, socio-economic class or sex. “The semi-structured interview guide was designed by the researchers in order to ensure it specifically met the aims of the study.”

### Data analysis

This research was conducted as a semi-structured, formative study and was not underpinned by a particular theory. Both inductive and deductive thematic analysis were used to avoid confirmation bias, yet acknowledging both the researcher and some participant’s had a diagnosis of asthma. Analysis was loosely connected to Braun and Clarke’s (2006) thematic analysis [6], which allowed flexibility in approach whilst still enabling the identification of themes in a robust and rigorous manner. It is hoped that a more comprehensive understanding of the perceptions of children around such topical issues as air pollution and physical activity, will enable a more successful approach to future interventions, with previous studies highlighting the need to consult would-be participants [23, 31]. Interviews were transcribed and initial analysis was completed immediately following the interview to establish dominant themes and enable the researcher to ascertain when data saturation was achieved. Data saturation was deemed to have occurred when no new themes were mentioned by children of either primary or secondary school age. Interview transcripts were read and examined several times by the first author to gain a more rounded understanding of the participant responses. Thematic analysis presented the opportunity for the identification and organisation of similar responses, which was conducted using QRS International’s NVivo 12 software. Concepts were then compared within and across the transcripts to enable the identification of global themes. Two separate authors critically examined the analysis as a means of triangulation, demonstrating methodological rigour and increasing the trustworthiness “criteria” of the research [32]. The responses were then presented in the form of pen profiles to determine the “strength” of each identified theme. Pen profiles provide a way of presenting the data in an easily accessible manner and allow for greater flexibility and creativity within qualitative research. This enables greater credibility and transferability [23]. Throughout the analysis, the potential impact of participant demographics on understanding and perceptions was explored separately.

## Results

**Fig. 1:**
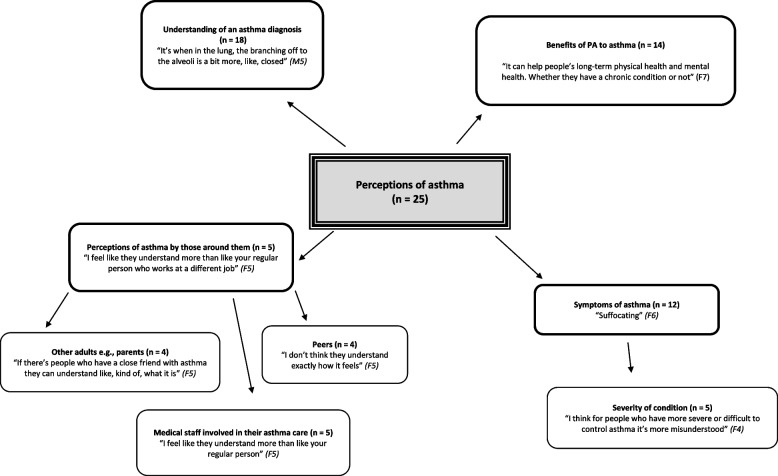
Children’s perceptions of asthma with quotes representative of their opinions. The strength of the themes are indicated by the number of children who identified each topic in discussion. The greater the number of times a topic was mentioned, for example “symptoms” or “friends”, the stronger the theme. Four key themes were identified: Understanding of an asthma diagnosis, benefits of PA to asthma, perception of asthma by those around them and symptoms of asthma

**Fig. 2:**
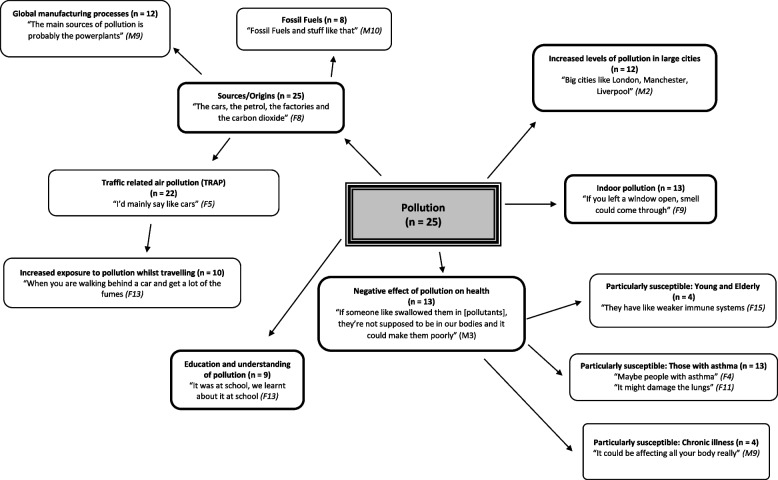
Children’s perceptions of the term “pollution”. The five key themes are highlighted, with the sub themes following. The strength of each theme was determined by the number of children who identified that topic within discussion. Key themes were Sources/Origins, education and understanding of pollution, negative effects of pollution on health, increased levels of pollution in large cities and indoor pollution

Participant (*n* = 25) quotes are labelled by sex (M = male, F = female) and age (in years). Key emergent themes were presented in the form of pen profiles, focusing on participant’s awareness of asthma (Fig. 1), as well as PA and pollution (Fig. 2). Four key emergent themes were identified relating to the participant’s awareness of asthma; namely understanding of an asthma diagnosis, benefits of PA to asthma, perceptions of asthma by those around them, and the symptoms of asthma (Fig. 1).

### Definitions and understanding of the key themes

The majority of children (72%) provided a reasonable description of the term asthma, mentioning the respiratory system and/or identifying the main symptoms of wheeze, cough and shortness of breath [15]. All children with asthma, with the exception of one seven-year-old child, were able to describe and explain the condition. Primary school aged participants without asthma identified the treatment for the disease – that is a ‘pump’ – but were unsure of what the condition itself was. Similarly, secondary school aged children identified “an inhaler” but were also unsure why, using phrases such as “I know some people use the pumps after exercise, but I don’t actually really know [what asthma itself is]” [F14, 17 years old, no asthma]. Asthma was often associated with exercise, with EIB being the identifying characteristic; referring to asthma being “something you get after exercise or sport” that means sufferers “can’t breathe properly and cough a lot” [F2, 11 years old, no asthma].

All children provided a description of what they understood about pollution and identified at least one source of air pollution. The majority of children (88%) indicated TRAP as a main source, with global manufacturing processes, such as the burning of fossil fuels (32%) identified by the younger children, whilst the production of greenhouse gases (12%) was highlighted by those in secondary school. There was no difference in understanding between those diagnosed with asthma and those without. Whilst all children identified the existence of pollution and poor outdoor air quality, only 52% demonstrated an awareness of indoor air pollutants. Of those who identified indoor pollution, eight (62%) had a diagnosis of asthma whilst 5 did not. The idea that pollution is a physical entity that can be touched or seen was alluded to in several of the youngest children’s interviews with comments such as “I don’t really know. I’ve never really seen it (pollution)” *[F9, 7 years old, no asthma].* When discussing outdoor air quality, 12 participants (48%) introduced the sub-theme of large cities, highlighting their belief that the larger the city, the poorer the air quality. This idea was further explored when the children spoke of their commute to school; of the 22 participants who described their commute, 10 made reference to an increase in traffic leading to the air feeling “foggy”. Of the 25 participants interviewed, 13 walked to school (52%), 11 (44%) travelled by car, and 1 (4%) rode a scooter. Those living in more deprived areas as well as those in secondary school more frequently reported travelling by car whereas those attending primary schools regularly walked.

Despite some uncertainty, all the children interviewed described pollution in a negative fashion, with 13 linking pollution to undesirable effects on health, specifically respiratory health; “I think it will affect the lungs. Maybe not noticeably, but it will still affect them” *[F11, 14 years old, no asthma].* Those with asthma were highlighted to be at an increased risk of the negative effects of pollution by 31% of participants, with both those with and without asthma identifying this association. Older adults and very young children were also identified as being vulnerable, with two participants describing a weakened immune system when rationalising their answer. Both these children attended secondary school.

Those with a diagnosis of asthma (*n* = 11; 7 girls, 4 boys) described their condition as “suffocating”, commenting “[my] lungs are kinda squeezed” [M8, 13 years old, asthma diagnosis]. Despite the tendency to describe asthma as purely related to exercise, 10 of the children identified asthma management strategies including PA. It should be noted that EIB and asthma are distinct entities, with EIB occurring in both asthmatic and non-asthmatic individuals [1]. It is possible, therefore, that the reported presence of EIB by children in this study does not actually represent ‘asthma’, but this is beyond the scope of this study. Specifically highlighted was the importance of pre-treatment before undertaking PA; “I just make sure that I’ve taken my blue [inhaler] before and don’t do too much” *[M6, 12 years old, asthma diagnosis].* However, five of the secondary school aged participants (all with a diagnosis or living with a close family member with asthma), alluded to the need to consider the severity of asthma when encouraging PA.

Physical activity was correctly identified as being associated with movement of the body by 22 (88%) of the children. However, almost all participants went on to mention physical education (PE) lessons at school, seeing PA and PE as interchangeable. When questioned further, secondary school aged adolescents were able to appreciate that they are physically active many times a day, not solely within these lessons, although younger children’s views were more restrictive.

The time the participants estimated they spent outside during the school day varied considerably from one to six hours a day. Alterations to the school day were mentioned by 18 participants (72%), with particular reference to an increase in the time spent outside, due to the COVID-19 pandemic. The wide variability in estimated times suggests this information may be too anecdotal and inaccurate to be included in the analysis.

### Education

The role of schools in the education of children on topics such as PA, pollution and asthma was also explored. All children received formal PE lessons at least once a week. Nine secondary school aged children remembered the topic of pollution being discussed at primary school. Those from secondary school credited their knowledge to geography lessons, whilst six children reported not having been taught formally. A lack of understanding regarding the long-term consequences of pollution on their health was also expressed by the older children who, when questioned, began to realise that “It [pollution] could be affecting your whole body really – your brain, it could be affecting your eyes. It’s quite a hard thing to pinpoint” *[M9, 14 years old, no asthma].*

### The influence of the environment on PA and health

Most children (92%) conveyed an element of confusion when the three separate topics were interlinked. All children agreed PA was beneficial, and pollution had a negative impact, but participants struggled when asked about the implications of being physically active in a polluted area. Socioeconomic status had no influence on the response, however, the answers became more complex with age. Eleven children (44%) believed that PA in a highly polluted area would be more dangerous to your health than beneficial, eight (32%) were unsure and suggested some form of compromise, whilst six (24%) believed the benefits gained would outweigh any danger to their health from poor air quality. Of those with asthma, 46% overall felt, PA in a polluted area would cause more harm than good, with only one children believing PA benefits would outweigh the negative aspects of pollution. Conversely, 64% of those without a diagnosis of asthma felt that if you were healthy, PA should still be encouraged regardless of air quality.

## Discussion

The primary aim of this study was to elicit the views of children and adolescents regarding PA, asthma and pollution and to explore any potential interaction between them. Overall, the participants understood the concept and importance of PA for health, with those with asthma also aware of its role within the management of the disease. Individuals with asthma showed a heightened awareness of the presence of indoor air pollution. All participants, irrespective of an asthma diagnosis, identified the presence of outdoor air pollution but some gaps were evident in the participants’ understanding of pollution, indoor air pollution and asthma, and their interaction. This is unsurprising given there is much debate around these topics in current research with research by Kim et al., [22] also finding indoor air pollution to be particularly poorly understood. Importantly, there was a clear willingness to enhance their understanding, highlighting the need, and opportunity, for an educational element in future interventions seeking to increase PA levels among children – particularly those with asthma.

The majority of participants were able to define the term pollution. However, even the adolescents struggled to develop a simple descriptor, often commenting “I don’t think anyone has ever sat down with me and talked to me about what it actually is…just what is affecting it” (F4, 17 years old, asthma diagnosis). The role of schools in the education of children was mentioned by nine of the 25 individuals interviewed, whilst only one child identified the media, suggesting that the recent campaigns for better air quality are not accessible to, or noticed by, children and adolescents. Most children were enthusiastic about the opportunity to increase their awareness, demonstrating that children have the desire to learn about environmental issues. The age at which children gain the level of cognitive development to conceptualise pollutants as invisible, yet present, particles requires further research. Some suggest children to be incapable of appreciating the closely woven interrelationships of environmental issues [4, 33] whilst others believe children conceptualise such scientific concepts in unique, idiosyncratic ways [9]. This study would tentatively support the later, with 88% of participants demonstrating an awareness of pollution, which could be utilised to introduce such concepts into the curriculum. However, some inaccuracies were noted regarding the understanding of ‘pollution’. Younger children often associated the term with smell, citing bonfire smoke, perfume or candles, suggesting young children do not differentiate a pollutant from an odour. This is in line with research by Bu et al., [7] who found children to use the term odour as proxy for dry air or poor indoor air quality. They determined that the presence of dry or irritating indoor compounds were found to be a surrogate for poor indoor air quality, which was positively correlated to the presence of asthma in young children.

Further confusion was evident with participants identifying solid waste as a source of air pollution, likely the result of the children looking to find a tangible representation for their idea of pollution. It would be beneficial to explore such idiosyncrasies further through other questioning techniques, such as drawing. Noonan et al., [25] through their “write, draw, show and tell” method determined that using a range of methods when working with children was not only more interactive but also more inclusive than traditional methodological approaches. This allowed for greater confidence in the results, as well as enhanced data credibility. Despite this, the ability to rationalise the problems pollution causes and identify solutions was mentioned by 76% of participants, with the concept of hybrid or electric vehicles being mentioned, or strategies such as ‘no parking outside of schools during school hours’ being suggested.

In exploring the children’s conceptualisations, only 13 children (52%) were able to acknowledge the existence of indoor pollutants. This concurs with Stevens et al., [36] who also reported some confusion when trying to recognise and distinguish indoor pollutants. Those diagnosed with asthma showed greater awareness of sources of indoor pollution (73%) than those without (27%), likely due to experiencing one or more ‘triggers’, such as aerosols, dust or smoke. This is supported by current research identifying ambient particulate matter as a significant contributor to poor asthma control [37], and the indoor environment being responsible for increased medication use and emergency hospital admissions [24]. The incorrect notion that indoor pollution is a product of something originating outside, that enters via windows and doors, was alluded to by several participants who believed outdoor pollution to be worse than indoor. Only one child correctly identified the need for good ventilation within the home. The identified misconceptions surrounding indoor and outdoor air pollution highlight the need for awareness campaigns to be targeted towards a greater age range, perhaps specifically targeting the younger population. Furthermore, for future interventions targeting behaviours based upon reducing exposure during PA to succeed, an educational arm is strongly advised to ensure children appreciate why such interventions are necessary. Without greater awareness, young people are unlikely to engage with intervention strategies post study, thus reducing clinical benefit.

In accordance with Fan et al., [12] 88% of the children interviewed in this study identified vehicle emissions as the main source of pollution and described a negative association with their respiratory health. Individuals in this study living in more densely populated areas, and those suffering from asthma, appeared more aware of TRAP than those living in more rural areas, with 91% believing it to negatively affect their asthma control. This concurs with recent meta-analysis by Khreis et al., [21] where a significant positive association was found between asthma and exposure to particulate matter. There is an increasing body of evidence linking air pollution, and in particular TRAP, to incidence of asthma and respiratory issues [26, 18, 13]. However, none of these studies have sought the opinions of, or understanding, of either parents or children.

More disparity was noted between participants’ perceptions of the lasting effects of pollution. Whilst some children (52%), most noticeably those with a diagnosis of asthma, believed poor air quality affected their health, others did not feel pollution was detrimental to their health. Specific groups perceived as being at an increased risk from exposure to pollution were those with lung disease or asthma (52%), the young and elderly (16%) and those with chronic health conditions (16%). All bar one participant was aged over 11 years, indicating an increased understanding of the deleterious effects of exposure to pollution with age. The children who identified at risk groups identified a weakened immune system and inability to fight off illness and ailments, suggesting a clear level of understanding. It is also worth noting that whilst 52% of participants highlighted those with asthma to be at greater risk, this may simply be the result of their awareness of the condition being a focus of the interview.

There is an abundance of research covering the importance and benefits of leading an active lifestyle, whilst simultaneously highlighting our failure to meet the recommended PA outcomes suggested by the WHO (2020) [40]. Despite understanding the importance of engaging in PA, the children interviewed rarely chose active behaviours during their free time. Sex and age appeared to play a central role, with boys, in accordance with Bean, Forneris & Fortier [5], reporting to engage in more active play than girls. However, contrary to Rota et al., [34] there was no suggestion by the participants in this study, that children with asthma were more sedentary than their healthy counterparts. Whilst research investigating the PA levels of youth with asthma remains equivocal, the current study provides further support to research by Winn et al., [39] which reported that 81% of those diagnosed with asthma identified engagement in PA as a favourite activity. Despite finding PA challenging, 91% of individuals with asthma engaged in PA as a means to control their condition. The potential interactive effect the severity of disease may have on PA levels was highlighted by three adolescents, all with asthma***.*** This suggests that more differentiated research in this area is needed when measuring PA among those with asthma and lends support to emerging research by Pike et al., [30] who acknowledge the role disease severity may play.

Few studies have explored young people’s perceptions of asthma, despite research suggesting that parents’ perceptions of asthma and PA are less reliable then their children’s [27]. It is also possible that the perceptions of children’s healthy peers may affect participation in PA. Similar gaps in the literature were highlighted by Winn et al., [39] who found limited evidence of studies that involved adolescents with asthma in study design. Indeed, 64% of children and adolescents with asthma in this study believed that their condition was difficult to comprehend and often misunderstood by others with one individual stating “unless you experience it yourself. It’s unknown to them and as many diagrams that they can use, you go through it. It’s not the same” [F6, 17 years old, asthma diagnosis]. Whilst the views shown by the healthy participants in this study suggest no misconceptions or negative opinions are held against those with asthma, whether real or perceived, addressing such ideas may improve the management of the condition leading to improved overall health.

One of the strengths of this study is the in part inductive, in part deductive approach, coupled with the use of pen profiles to present the data. This enables the reader to gain a diagrammatic overview of key emergent themes clearly and succinctly. Analysis and presentation of qualitative data in such a manner not only limits the analysis being skewed by key dominant participants, but presents the data in a more objective manner [23]. Due to the current COVID-19 pandemic, in-person interviewing was not feasible, but video interviews over Zoom provided both a time- and cost-effective solution. The increased accessibility removed many logistical factors, such as geographical location, meaning we were able to reach a wider number of individuals [16]. However, participant recruitment also relied heavily on social media, thus determining the demographic of volunteers and limiting those able to participate to those with access to a computer and the internet.

Whilst there are numerous strengths associated with this study, several limitations are noteworthy. In any situation where individuals put themselves forwards an element of self-selection bias may be present. There is also the possibility of social desirability with participants giving the answer they believe the researcher wishes to hear, although the interviewer made sure to emphasise that there were no right or wrong answers. It is also beyond the scope of the current study to explore the influence of factors such as sex, ethnicity and socioeconomic status on barriers and facilitators, and their interaction with pollution, although this does warrant further investigation. Specifically, some participants were unwilling to share their home postcode, limiting our insights to participant’s socio-economic status. Whilst an inclusive approach to recruitment was taken, the subsample (88% of the total sample) of socio-economic data we were able to collect suggested a predominantly upper quartile bias to the SES of the participants. It is therefore possible that the opinions of the participant’s in this study are influenced by a higher literacy ability, as has been previously shown [19], and the generalisability of the findings to other SES quartiles should be considered. Future research should also seek to integrate parental views, which may help determine how, when and why children develop the perceptions they do.

## Conclusion

This study suggests that children and adolescents are both capable of understanding the deleterious effects of poor air quality as well as the unknown long-term cumulative effects of exposure to pollutants and showed a willingness to further their understanding. The enthusiasm shown by the participants in this study show that children and adolescents are capable of, and indeed keen to, learn about the topics of air pollution and PA and how these may aspects of their health. Including an educational arm to future interventions would reduce misconceptions and ensure informed decisions are made regarding PA and how this may be affected by pollution and health status. We also recommend approaching air pollution with primary school aged children who have shown a willingness and capability to understand these topics. Strategies such as these will be particularly pertinent for youth with asthma, who already recognise poor air quality to negatively affect disease control.

## Data Availability

The data generated during this current study is not publicly available in order to maintain confidentiality, but are available from the corresponding author on reasonable request.

## Abbreviations

DEFRA: Department for environment, food and rural affairs
EIB: Exercise Induced bronchoconstriction
FeNO: Exhaled Nitric oxide
GINA: Global Initiative for asthma
PA: Physical activity
PE: Physical Education
SES: Socio-economic status
TRAP: Traffic related air pollution
WHO: World Health Organisation
